# Menopause in nonhuman mammals—What does it mean for the gynecologist?

**DOI:** 10.1111/aogs.70131

**Published:** 2025-12-21

**Authors:** Sebastian Gidlöf, Hedvig Engberg, Ivika Jakson

**Affiliations:** ^1^ Department of Clinical Science, Intervention and Technology Karolinska Institutet Stockholm Sweden; ^2^ Department of Women's and Children's Health Karolinska Institutet Stockholm Sweden; ^3^ Medical Unit Gynecology and Reproductive Medicine Karolinska University Hospital Stockholm Sweden

Menopause has historically been regarded as a specific human phenomenon, but new research in comparative and evolutionary biology suggests that reproductive aging and reduced ovarian hormone production are not entirely unique to humans.

## 1 SPECIES

It has previously been shown that some species of toothed whales display a clear postreproductive life phase meaning that reproduction is haltered well before death.^1^ Toothed whales represent the first of the two living groups of cetaceans and include species like dolphins, killer‐whales, beluga, etc. The other group of cetaceans includes whales that have baleen instead of teeth, such as blue whales, gray whales, humpback whales, etc. Walker and Herndon^2^ reviewed the topic and found that although some nonhuman primates seem to encounter menopause, the most compelling evidence comes from whales.

Later reports have shed new light and a study in wild populations of chimpanzees (*Pan troglodytes*) found evidence for a postreproductive phase in females. However, this phase does not seem to have such a sharp onset as menopause in the human sense.^3^

Studies in the African elephant (*Elaphas maximus*) suggest that many females live many years after their last reproduction even though fertility in this species seem to gradually decline over several decades rather than ending within a few years.^4^

The term *menopause*, referring to the last menstruation in life, is actually misleading as only a few mammals do have a menstrual cycle. Winkler and Goncalves have therefore advocated the use of *oopause* to allow for better comparison between mammal species.^5^ Such a definition would include a broader understanding of the permanent cessation of ovulations, which may be more common in mammals than we have previously thought.

## 2 SIGNS, SYMPTOMS AND PHYSIOLOGICAL CHANGES

In humans, the perimenopause surrounding menopause is accompanied not only by the cessation of ovulations but also hormonal changes (declining levels of estrogen and progesterone and the reciprocal elevation of gonadotrophins), symptoms (such as hot flushes and vaginal atrophy), and physical changes (reduced bone mineral density and cardiovascular changes). Data from other species are scarce.

Studies from toothed whales are based on demographics, morphology, and lifespan markers. Photopoulou et al. found morphological signs of ovarian inactivity in older individuals of false killer whales (*Pseudorca crassidens*). By comparing the age of the whales to ovarian morphology, they could draw the conclusion that the species displays a postreproductive life phase.^6^

In chimpanzees, evidence is based on behavioral studies, demographic data, and hormonal analyses. In the Ngogo population in Uganda, females older than 50 years did not have any offspring, and hormonal urinary samples indicated reduced ovulatory cycling.^3^

The decline in elephants is gradual and fertility starts to decline at above 50 years of age. Still, many female individuals continue to reproduce well into their 60s. There is thus no distinct upper limit.^4^

Symptoms corresponding to hot flushes, mood disturbances or other climacteric symptoms are notoriously difficult to study in wild mammals. The focus has therefore been on reproductive measurements such as age at last birth, signs of ovulation, and postreproductive survival.

Experimental studies in laboratory animal models (such as oophorectomized rodents or chemically induced follicle depletion) skip the natural situation of menopause or oopause and do not take the life history perspective into account.

## 3 POTENTIAL ADVANTAGES

Why would a female individual cease reproduction long before death? The theory of evolution demands answers and many hypotheses have been put forward.

In toothed whales, the *grandmother hypotheses* have strong evidence. Ellis et al. found that menopause in toothed whales was developed when female lifespan was prolonged without the reproductive period doing so. This increased the gap between generations and made co‐operation, transfer of knowledge on such important aspects as hunting and migration possible as well as reducing the reproductive conflict between generations.^1^ This theory fits with the species' natural history where females stay in the family group with their mothers their whole life, developing matrilineal pods. By stopping reproduction, the older mother avoids competition with her daughters and at the same time contributes to the survival of the whole family. In an evolutionary sense, the postreproductive female improves the evolutionary fitness of the species and increases the chances of her genes to be conveyed to further downstream generations.

In addition, studies in chimpanzees suggest the fact that certain females live many years after their last birth and that older age may give advantages such as knowledge and care, although the social structure is different from that of whales, making the grandmother effect somewhat weaker.^3^

Elephants also share a grandmother‐like structure but the absence of a distinct menopause indicates that the advantages of continuing reproduction in older age may outweigh the costs. Fertility declines gradually but reproduction does not stop at a certain time. In a study that compared preindustrial humans with elephants, Lahdenperä and co‐workers conclude that even though some species develop a long lifespan, they do not necessarily develop menopause.^4^

Thus, the advantages of menopause, oopause or post‐reproductive life phase seem to include the following:

1. *Support for genetic family members over generation gaps*—older females contribute to offspring and grand‐offspring survival and thereby their possibility to reproduce.
2. *Avoiding reproductive generational conflicts*—by ceasing to reproduce older females avoid competition with their daughters and possibly also competition between offspring and grand‐offspring.
3. *Life‐span advantages*—in species with long lifespan, reproduction in old age may be risky, and to stop reproducing may be more adaptive, in an evolutionary sense, increasing the chances of conveying alleles by increasing the chance of offspring and grand‐offspring survival.

## 4 IMPLICATIONS FOR HUMANS AND CONCLUSIONS

### 4.1 Rarity suggests specific life history contexts

The fact that menopause in a strict meaning (a sharp reproductive end followed by a long survival) is rare among mammals suggests that its evolution depends on specific social and life history conditions. Toothed whales represent one model, humans another.

Data from elephants show that long lifespans are not enough and that social structure and herd dynamics play crucial roles. In humans, the evolutionary roots of menopause may lie in our unique social structures with long childhood dependence, prolonged lifespan, close matrilineal lineage and grandmother support. Life expectancy at birth was very low before modern times because high infant mortality pulled down the average, even though those who survived to reproductive age lived much longer. This implies that only individuals who reached reproductive age passed on their genes and that many women lived long enough to reach menopause, supporting the idea that grandmothers could improve their grandchildren's survival chances.

### 4.2 The value of the postreproductive female

The examples from other species support the idea that a postreproductive life phase may be evolutionary adaptive by help from older relatives rather than a random side‐effect of a long life. In humans, the grandmother theory is old and studies in toothed whales support its evolutionary background. This suggests that menopause and a long life after fertility improves the evolutionary fitness of the species.

### 4.3 Physiological versus life history mechanisms

Comparative data show that menopause is not inevitable in all long‐lived mammals. The physiological cessation of ovulation probably requires both follicle depletion and a certain evolutionary selection. This raises questions also for human physiology. Perhaps humans, like toothed whales, have developed a long postreproductive life phase where the drastic hormonal change is the price to pay for ascertaining the well‐being of grand‐offspring.

### 4.4 Re‐thinking symptoms and biology

Even though wild animals do not report hot flushes or mood disturbances, the fact that ovarian and endocrine changes follow reproductive aging in several species suggests human climacteric partly mirrors symptoms also in other species. Laboratory animal models are useful but lack the evolutionary context with natural aging. More long‐term field studies may cast light on natural menopause and oopause in wild nonhuman mammals.

## 5 IMPLICATIONS FOR HEALTH AND AGING

To understand why older nonhuman females stop to reproduce but continue to live on may provide knowledge also stretching further than perimenopause. A long postreproductive life‐phase means that body maintenance (brain, bone, heart, etc.) is an evolutionary trait selected to work for decades after fertile age.

This provides both hope and challenge. It suggests that intervention should focus not only on restoring or mimicking ovarian function but also on the whole organism's overall long‐term health. This means that menopause should not be regarded as only a loss of fertility and ovarian function but as a transition from one evolutionary function to another.

## 6 FINAL REFLECTIONS

In conclusion, even though menopause in a strict human sense is rare in the animal kingdom, comparative biology studies suggest that in nonhuman mammals, in particular toothed whales and, more recently described, chimpanzees, a postreproductive life phase can develop under certain species‐specific life history conditions. The absence of a distinct oopause in elephants underpins that a long lifespan is not enough and that other evolutionary selective factors such as intergenerational co‐operation are crucial.

In humans, this supports the understanding that menopause is deeply embedded in our species' social and evolutionary history. Rather than being a side effect of a long lifespan, it is shifting from one evolutionary phase to another. A more integrated understanding, including physiology as well as social factors, may lead to a deeper understanding and possibly also better support of women undergoing perimenopause.

## AUTHOR CONTRIBUTIONS

S.G. wrote the first draft, which was revised by H.E. and I.J.

## Data Availability

Research data are not shared.
